# Fatal Pediatric Metformin-Associated Lactic Acidosis: When Severity Goes Unnoticed

**DOI:** 10.7759/cureus.103719

**Published:** 2026-02-16

**Authors:** Inês Noites, Marta Figueiredo, Zakhar Shchomak, Leonor Boto, Cristina Camilo

**Affiliations:** 1 Pediatric Intensive Care Unit, Pediatrics Department, Unidade Local de Saúde de Santa Maria, Lisbon, PRT; 2 Pediatrics Department, Hospital do Divino Espírito Santo de Ponta Delgada, Ponta Delgada, PRT; 3 Pediatrics Department, Unidade Local de Saúde de Lisboa Ocidental, Lisbon, PRT

**Keywords:** brain death, hypoglycemia, metformin-associated lactic acidosis, metformin overdose, pediatric intensive care unit

## Abstract

Metformin poisoning is rare in the pediatric population but can lead to life-threatening metformin-associated lactic acidosis (MALA) and multiorgan failure, particularly when diagnosis or intervention is delayed. We report the case of a 16-year-old girl who intentionally ingested 34 g of her prescribed metformin following an emotional trigger. She presented to the emergency department one hour after ingestion, hemodynamically stable with unremarkable laboratory findings; however, venous blood gas analysis was not performed at admission. Within 15 hours, she developed severe metabolic acidosis consistent with MALA, acute kidney injury, and progressive hemodynamic instability requiring resuscitative efforts, mechanical ventilation, and vasopressor support. Continuous renal replacement therapy was initiated in the pediatric intensive care unit, but no meaningful clinical improvement was observed. Despite aggressive management, she developed cerebral edema with cerebellar tonsillar herniation and was declared brain dead 46 hours after ingestion. This case reinforces the importance of early recognition and prompt multidisciplinary management of patients at high risk for MALA, given the potential for rapid progression to severe and refractory metabolic acidosis, even in individuals who appear clinically stable at presentation. It also highlights hypoglycemia as a rare but critical manifestation of severe metformin toxicity.

## Introduction

Metformin, a biguanide commonly used to manage type 2 diabetes and insulin resistance, is increasingly prescribed to pediatric patients with obesity-related insulin resistance. While generally considered safe at therapeutic doses, metformin overdose can cause life-threatening complications, including severe lactic acidosis, electrolyte imbalances, and multiorgan failure, particularly in the setting of renal dysfunction [1-3]. In rare cases, high-dose ingestion may also precipitate hypoglycemia, especially in individuals with poor nutritional status or underlying metabolic disorders [4].

Metformin-associated lactic acidosis (MALA) is an uncommon but severe condition with a reported incidence of approximately 4.3 cases per 100,000 patient-years and a mortality rate ranging from 20% to 36% [2,5]. While most fatal cases have been reported in adults, pediatric cases have also been described, primarily following intentional ingestions [1,2,6]. Toxic effects in children have been observed at doses exceeding 100 mg/kg [7]. Early symptoms are often non-specific, including nausea, vomiting, abdominal discomfort, malaise, or mild drowsiness, which can delay diagnosis and timely intervention [1,7].

We report a fatal case of MALA in an adolescent patient, highlighting the critical role of early recognition and prompt multidisciplinary management.

## Case presentation

A 16-year-old adolescent female with a history of obesity, binge eating disorder, and insulin resistance was being treated with metformin 850 mg once daily. She weighed 107 kg and had no personal history of self-harming behaviors or previous episodes of intentional drug intoxication. Following an emotionally distressing event, she impulsively ingested 40 tablets of metformin, corresponding to a total dose of 34 g (approximately 318 mg/kg).

She presented to the pediatric emergency department one hour after ingestion with a Glasgow Coma Scale (GCS) score of 14. She was hemodynamically stable, mildly nauseated, and visibly distressed, with no focal neurological deficits or signs of respiratory compromise. After consultation with a national poison control center, gastric lavage was performed, and activated charcoal was administered via nasogastric tube.

Initial laboratory tests (Table 1) revealed mild leukocytosis, blood glucose of 181 mg/dL, and mildly elevated C-reactive protein (30 mg/L). Renal and liver function tests, as well as serum electrolytes, were within normal limits. Venous blood gas analysis was not performed at admission.

**Table 1:** Main laboratory test results *Survivor's gasimetric severity thresholds: pH > 6.9, lactate < 25 mmol/L. Values outside these ranges (pH < 6.9 or lactate > 25 mmol/L) indicate severe metabolic derangement and correlate negatively with survival. PICU: paediatric intensive care unit, INR: international normalised ratio, aPTT: activated partial thromboplastin time.

Analytical parameters	1 hour post-ingestion	16 hours post-ingestion	Pre-transfer	PICU	Reference interval
Gasimetry					
pH*	–	6.73	6.55	7.13	7.35–7.45
Bicarbonate (mmol/L)	–	4.3	4.2	21.5	22–26
Lactate (mmol/L)*	–	26	26	31.5→1.8	0.50–2.00
Renal function					
Urea (mg/dL)	21	27	–	30→35	15–40
Creatinine (mg/dL)	1.0	2.63	–	3.03→1.83	0.5–1.0
Hematology					
Leukocytes (/μL)	15,300	–	44,800	49,400→18,400	5,000–15,000
Liver & muscle enzymes					
Aspartate aminotransferase (U/L)	30	128	245	308	<40
Alanine aminotransferase (U/L)	40	106	200	264	<45
Cardiac markers					
Creatine kinase (U/L)	–	–	1,813	11,893	<170
Inflammatory markers					
C-reactive protein (mg/L)	30.3	–		100.9	<5
Coagulation					
INR	1.0	–	4.63	4.6→1.38	Varies according to condition
aPTT (s)	28	–	82.4	49→27	25–35

Over the subsequent 15 hours, the patient developed watery diarrhea and experienced three documented episodes of hypoglycemia, with a nadir blood glucose of 39 mg/dL. Approximately 16 hours after ingestion, repeat laboratory evaluation revealed acute kidney injury (urea 27 mg/dL; creatinine 2.63 mg/dL) and severe metabolic acidosis with hyperlactatemia (pH 6.73, HCO₃⁻ 4.3 mmol/L; lactate 26 mmol/L). Pediatric intensive care consultation was requested, and the local transport team was activated for transfer.

Shortly thereafter, her condition deteriorated, with progressively worsening depression of consciousness, hypotension, and increasing respiratory distress. Initial resuscitative measures included intravenous fluid boluses and sodium bicarbonate. Progressive bradycardia (59 bpm) and hypotension (70/36 mmHg) prompted administration of atropine, placement of central venous access in the left jugular vein, and initiation of inotropic support with dopamine (10 μg/kg/min). Repeat laboratory testing revealed coagulopathy (INR 4.63; aPTT 82.4 s) and worsening metabolic acidosis (nadir pH 6.55; HCO₃⁻ 4.2 mmol/L).

Upon arrival of the transport team, she was profoundly bradycardic (heart rate 30 bpm) with unmeasurable blood pressure, despite bag-mask ventilation. She was intubated, and cardiopulmonary resuscitation was initiated, requiring administration of two doses of epinephrine. Return of spontaneous circulation was achieved after six minutes. Bedside echocardiography revealed preserved biventricular function. Dopamine was discontinued, and norepinephrine (0.41 μg/kg/min) was initiated, resulting in transient hemodynamic improvement.

At admission to the pediatric intensive care unit (PICU), the patient was comatose (GCS 3) and mechanically ventilated. Pupils were initially reactive, and invasive arterial monitoring revealed low but measurable blood pressures. Laboratory evaluation confirmed multiorgan dysfunction, including worsening renal failure (urea 30 mg/dL; creatinine 3.03 mg/dL), persistent coagulopathy (INR 4.6; aPTT 49 s), and lactic acidosis initially exceeding the assay’s measurable range, with the highest upper limit of quantification at 31.5 mmol/L.

Continuous venovenous hemodiafiltration (CVVHDF) was initiated due to severe and refractory metabolic acidosis and acute kidney injury. Dialysis parameters were titrated based on serial laboratory results. Despite partial biochemical improvement, including reductions in lactate (1.8 mmol/L) and creatinine (1.83 mg/dL), no meaningful clinical recovery occurred. Transcranial Doppler assessment revealed no signs of intracranial hypertension.

Hemodynamic instability persisted throughout the PICU stay, requiring escalating vasopressor and inotropic support, including norepinephrine (max. 1.5 μg/kg/min), epinephrine (max. 0.2 μg/kg/min), vasopressin (0.03 U/min), and hydrocortisone (100 mg loading dose, followed by maintenance of 50 mg every 6 hours, totaling 200 mg per day). Rhabdomyolysis (max. CK 11,893 U/L) and hypertransaminasemia (ALT 264 U/L; AST 308 U/L) developed, with elevated inflammatory markers (max. leukocytes 49,400/μL; C-reactive protein 100.9 mg/L; PCT 7.87 ng/mL). Broad-spectrum antibiotics (piperacillin-tazobactam 4.5 g every 8 hours) were empirically started.

Later that day, the patient developed focal myoclonic seizures involving the left hemiface and lower limb, which progressed to tonic-clonic generalized seizures. The seizures were initially treated with benzodiazepines and a propofol bolus, followed by a continuous midazolam infusion (max. 1.4 μg/kg/min).

Approximately 36 hours after ingestion, she was noted to have fixed and dilated pupils. Cranial computed tomography revealed diffuse cerebral edema with multiple bilateral deep gray matter and cortical hypodensities, consistent with hypoxic-ischemic and hypoglycemic encephalopathy, as well as signs of transtentorial herniation. Repeat transcranial Doppler demonstrated the absence of cerebral blood flow. Brain death was confirmed 46 hours post-ingestion according to international guidelines, and the family consented to organ donation.

## Discussion

This case illustrates the severe and often underestimated risks associated with metformin overdose in pediatric patients. Despite initially normal clinical and laboratory findings, the patient experienced rapid deterioration, ultimately progressing to multiorgan failure and brain death. This clinical course highlights the critical importance of early metabolic assessment, particularly blood gas analysis, in suspected metformin toxicity, as profound metabolic derangements may be present despite an apparently benign initial presentation. In this case, the absence of an early gasometric evaluation may have delayed recognition of the underlying severe metabolic acidosis and postponed timely escalation of care.

Although pediatric metformin overdose is uncommon, its use is increasing among adolescents for obesity-related insulin resistance, warranting heightened clinical awareness. MALA is characterized by profound metabolic acidosis, markedly elevated lactate levels, and evidence of organ hypoperfusion. Early clinical signs may be subtle or nonspecific, and rapid deterioration can occur within hours of ingestion [1,5,7].

Hypoglycemia is an uncommon but recognized manifestation of massive metformin overdose, particularly in the context of prolonged fasting, underlying comorbidities, or impaired hepatic metabolism [4,8].

Hemodialysis is the preferred method for drug clearance in cases of severe metformin toxicity, owing to the drug’s low molecular weight, negligible protein binding, and large volume of distribution. Current clinical guidelines recommend initiating extracorporeal treatment when lactate exceeds 20 mmol/L, pH falls below 7.0, or when there is no improvement after 2 to 4 hours of supportive care [3,7]. In pediatric patients, continuous modalities such as CVVHDF are often preferred due to better hemodynamic stability, especially in the setting of vasoplegic shock, a common presentation in MALA [7].

Similar pediatric cases of metformin overdose requiring renal replacement therapy have been reported, with outcomes varying based on the timing of intervention and the presence of comorbidities [1-3,6,7]. However, cases resulting in confirmed brain death remain exceedingly rare. This report contributes to the growing body of evidence emphasizing the importance of early critical care involvement, even in patients who appear stable on initial assessment. A systematic review found that survivors of severe metformin toxicity typically present with a pH >6.9 and maximum lactate levels <25 mmol/L, highlighting the prognostic significance of early laboratory markers [9].

In this case, acid-base status was not evaluated at the initial presentation, potentially delaying recognition of the severity of toxicity. Earlier transfer to intensive care and prompt initiation of renal replacement therapy might have altered the clinical course.

This report highlights the need for a high index of suspicion in pediatric metformin ingestion, as massive overdose can appear clinically stable initially but may progress rapidly to multiorgan failure and brain death. Continuous monitoring of acid-base status, lactate levels, blood glucose, and renal function is essential. Early coordination with toxicology, nephrology, and intensive care teams should be strongly considered to guide timely and effective management. Prompt supportive care and consideration of extracorporeal therapies are critical, even in patients presenting with mild or nonspecific symptoms.

## Conclusions

MALA in adolescents, although rare, can progress rapidly and unpredictably, even when initial clinical and laboratory findings appear reassuring. This case illustrates the potential for massive overdose to cause multiorgan failure, severe metabolic derangements, and fatal neurological outcomes. Early recognition, close monitoring of acid-base status, lactate, blood glucose, and renal function, and timely multidisciplinary intervention, including consideration of extracorporeal therapies, are essential to prevent fatal outcomes and minimize long-term sequelae. Clinicians should maintain a high index of suspicion for MALA in any pediatric patient with significant metformin ingestion, as prompt recognition, aggressive management, and timely escalation of care may be lifesaving.
